# Supplementary material for: Tetrapods on the EDGE: Overcoming data limitations to identify phylogenetic conservation priorities
Source: PLoS One. 2018 Apr 11;13(4):e0194680. doi: 10.1371/journal.pone.0194680 (PMC5894989; doi:10.1371/journal.pone.0194680)
Supplement: S1 Text — (DOCX) [file pone.0194680.s001.docx]

**S1. Supplementary material**

To test the assumption that species with complete genera and families have the most robust ED scores, we followed five steps: 1) ‘reference’ ED and EDGE scores were calculated from consensus phylogenies for amphibians [1], crocodilians [2], testudines [3], and squamates (the older, Zheng and Wiens phylogeny, [4]); 2) a random number of species were removed from each phylogeny and the ED and EDGE scores of the remaining species were calculated; 3) the ED and EDGE scores from step 2 were compared to the ‘reference’ scores from step 1 by calculating the proportional change for each species (i.e. re-calculated ED divided by reference ED); and 4) each species was identified as either having a) a complete genus and family or b) incomplete genus and family remaining in the phylogeny after the random removal of species. We considered complete genera and families to be those that retained all species present in the published phylogeny before random removal.

Stages 2 to 4 were repeated 100 times, to give a range of values for the proportional change in ED and EDGE for each species when it had both a complete and incomplete genus and family, from which we calculated a median value for both scenarios for each species. Using the paired values for each species, we then ran a Wilcoxon signed rank test to test if the proportional change for each species was lower when it had complete genus and family versus when it had an incomplete genus and family. We combined data from all taxonomic groups. If our assumption was valid, we would expect to find that, following the artificial removal of species, ED/EDGE scores calculated for species with complete genera/families would experience significantly lower levels of overestimation than the ED/EDGE scores of species with missing congeners/family members.

We found that the overestimation of ED and EDGE scores of individual species for which all congeners or family members are present was indeed significantly lower than that of the scores for species with absent congeners or confamilials (Fig 1). Median overestimation of ED was 0.7% and overestimation of EDGE was 8.9% when the entire genus was present in the phylogeny, both of which were significantly higher when congeners were absent (overestimation of ED: 21.4%, EDGE: 39.5%) (Wilcoxon Signed-rank test, **ED:** V = 1.14×10^6^, Z = -33.68, d.f. = 7,657, p < 0.0001; **EDGE:** V = 1.01×10^6^, d.f. = 3,871, p < 0.0001). When considered at the family level, median overestimation of ED was 0.2% and overestimation of EDGE was 8.1% when the entire family was present in the phylogeny, both of which were significantly higher when confamilials were absent (overestimation of ED: 23.4%, EDGE: 12.4%) (Wilcoxon Signed-rank test, **ED:** V = 64,703, Z = -16.30, d.f. = 7,657, p < 0.0001; **EDGE:** V = 1.013.51×1058, d.f. = 3,871, p < 0.0001).

These results therefore support our assumption that species with all congeners and family member in the tree have more robust ED scores than those without. Thus, when using single consensus phylogenies to calculate ED scores we consider top 100 ranked EDGE species for which the entire genus or family is present in the phylogeny as robust priority species.

**Figure**


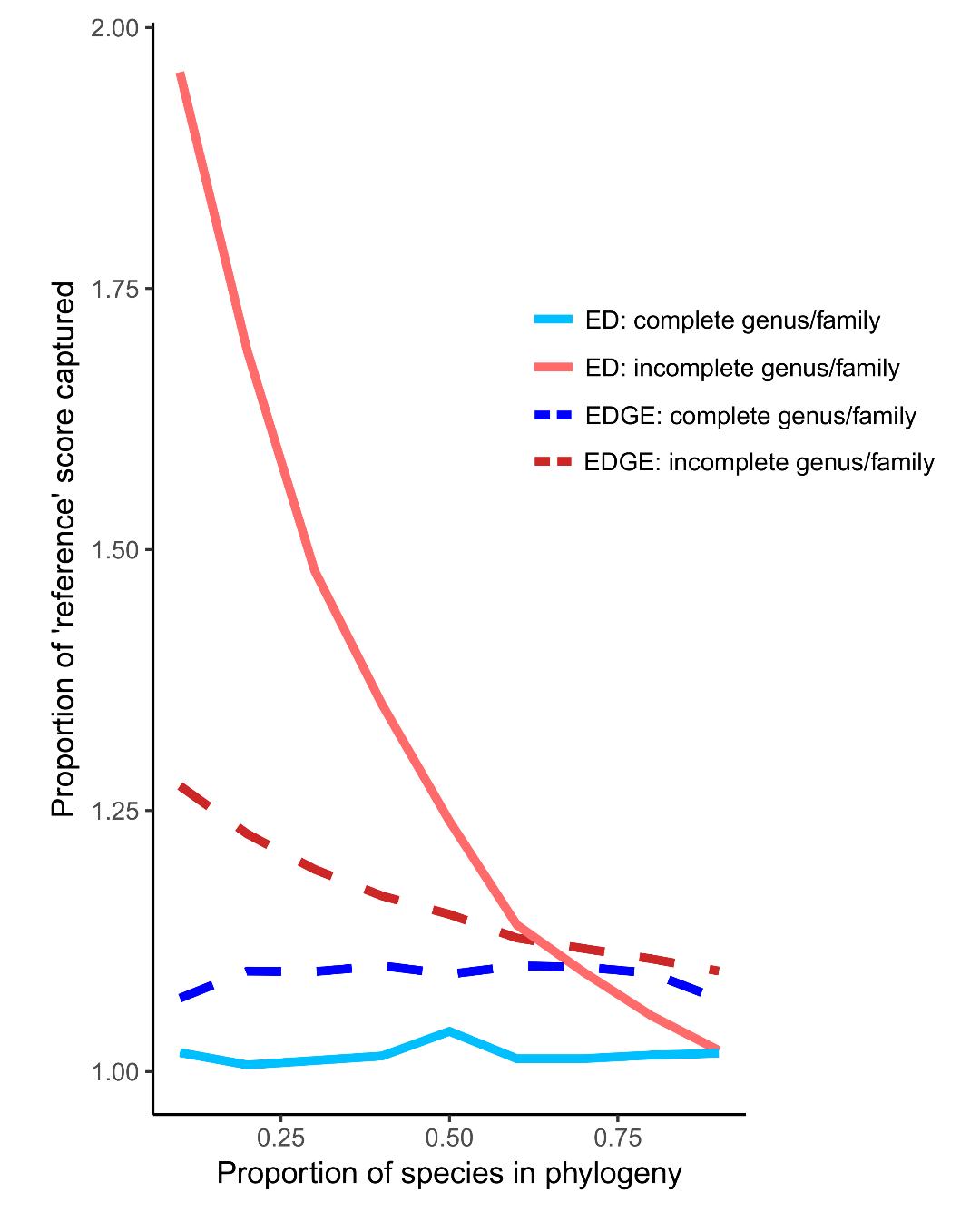


**Figure 1:** **Relative overestimation of ED and EDGE scores of species in phylogenies that are missing species but either contain or omit congeners and family members.**

Proportion of ‘reference’ ED and EDGE captured for species when their entire genus or family is either present (blue lines) or absent (red lines) from the phylogeny. Solid lines represent median overestimation of ED, and dashed lines represent median overestimation of EDGE, when phylogenies are degraded from 100% to 10%. Proportion of ED and EDGE captured is always an overestimation (i.e. greater than ‘reference’ ED/EDGE) as the ED of each species can only increase as others are removed.

**References**

1. Pyron RA. Biogeographic analysis reveals ancient continental vicariance and recent oceanic dispersal in amphibians. Syst Biol. 2014;63: 779–797. doi:10.1093/sysbio/syu042

2. Shirley MH, Vliet KA, Carr AN, Austin JD. Rigorous approaches to species delimitation have significant implications for African crocodilian systematics and conservation. Proc R Soc B. 2014;281: 20132483. doi:10.1098/rspb.2013.2483

3. Hedges SB, Marin J, Suleski M, Paymer M, Kumar S. Tree of life reveals clock-like speciation and diversification. Mol Biol Evol. 2015;32: 835–845. doi:10.1093/molbev/msv037

4. Zheng Y, Wiens JJ. Combining phylogenomic and supermatrix approaches, and a time-calibrated phylogeny for squamate reptiles (lizards and snakes) based on 52 genes and 4162 species. Mol Phylogenet Evol. Elsevier Inc.; 2016;94: 537–547. doi:10.1016/j.ympev.2015.10.009
